# Coevolution of the ATPase ClpV, the Sheath Proteins TssB and TssC, and the Accessory Protein TagJ/HsiE1 Distinguishes Type VI Secretion Classes*

**DOI:** 10.1074/jbc.M114.600510

**Published:** 2014-10-10

**Authors:** Andreas Förster, Sara Planamente, Eleni Manoli, Nadine S. Lossi, Paul S. Freemont, Alain Filloux

**Affiliations:** From the ‡ Centre for Structural Biology and; the § Medical Research Council Centre for Molecular Bacteriology and Infection, Department of Life Sciences, Imperial College London, London SW7 2AZ, United Kingdom

**Keywords:** ATPases Associated with Diverse Cellular Activities (AAA), Bacteriophage, Protein Evolution, Protein Secretion, Pseudomonas aeruginosa (P. aeruginosa), ClpV, Type VI Secretion System (T6SS)

## Abstract

The type VI secretion system (T6SS) is a bacterial nanomachine for the transport of effector molecules into prokaryotic and eukaryotic cells. It involves the assembly of a tubular structure composed of TssB and TssC that is similar to the tail sheath of bacteriophages. The sheath contracts to provide the energy needed for effector delivery. The AAA^+^ ATPase ClpV disassembles the contracted sheath, which resets the systems for reassembly of an extended sheath that is ready to fire again. This mechanism is crucial for T6SS function. In *Vibrio cholerae*, ClpV binds the N terminus of TssC within a hydrophobic groove. In this study, we resolved the crystal structure of the N-terminal domain of *Pseudomonas aeruginosa* ClpV1 and observed structural alterations in the hydrophobic groove. The modification in the ClpV1 groove is matched by a change in the N terminus of TssC, suggesting the existence of distinct T6SS classes. An accessory T6SS component, TagJ/HsiE, exists predominantly in one of the classes. Using bacterial two-hybrid approaches, we showed that the *P. aeruginosa* homolog HsiE1 interacts strongly with ClpV1. We then resolved the crystal structure of HsiE1 in complex with the N terminus of HsiB1, a TssB homolog and component of the contractile sheath. Phylogenetic analysis confirmed that these differences distinguish T6SS classes that resulted from a functional co-evolution between TssB, TssC, TagJ/HsiE, and ClpV. The interaction of TagJ/HsiE with the sheath as well as with ClpV suggests an alternative mode of disassembly in which HsiE recruits the ATPase to the sheath.

## Teaser

**Background:** The bacterial type VI secretion apparatus is a phage tail-like structure that secretes toxins/effectors.

**Results:** Four components of the type VI secretion system have coevolved and interact.

**Conclusion:** Distinct classes of type VI secretion systems emerge from the phylogenetic data.

**Significance:** Molecular understanding of type VI secretion might make it amenable to targeting by antimicrobials.

## Introduction

Gram-negative bacteria have evolved various strategies to compete in hostile environments. Among them, secretion systems have attracted a lot of attention because of their clinical importance but also because of their complex architecture and regulation. The conserved bacterial type VI secretion system (T6SS)^5^ (1, 2) was initially associated with bacteria-host interaction (3–6) but subsequently found to inject toxins into bacterial targets (7–12). These toxins have various activities, the best characterized of which are amidases (13), phospholipases (12), and nucleases (14). However, the list of T6SS toxins and their cognate immunity is expanding (15, 16), whereas their role in bacterial competition during host colonization is beginning to be established (14, 17).

A striking feature of the T6SS is its homology to the tail of the T4 phage. The gp19 tail tube is the conduit through which the phage DNA is injected, whereas the gp5-gp27 heterotrimeric complex is the puncturing device that penetrates the host cell membrane (18). The force needed to push the tube and puncturing device is provided by the contraction of the gp18 sheath (19). In the T6SS, TssB and TssC oligomerize into a sheath-like structure, similar to gp18 (20–24), whereas VgrG and Hcp share features with the puncturing device, gp5-gp27, and the tail tube, gp19, respectively (25).

A fundamental distinction between phage and T6SS is the dynamic nature of injection. The bacteriophage sheath contraction is a singular event, which empties the phage head of its DNA. In this case, return to the original state is unnecessary, and no energy is required to reset the system. In contrast, in T6SS, a series of assembly and contraction events of the TssB/TssC sheath are observed (20, 26, 27), which result in several bursts of toxin injection. ClpV, an AAA^+^ (ATPase associated with various cellular activities) protein, provides energy for sheath disassembly (21, 27, 28).

AAA^+^ proteins are hexameric ring-shaped complexes involved in a variety of functions, including protein quality control (29). In the T6SS, ClpV is proposed to disassemble the contracted sheath. In support of this model, it was shown that a *Vibrio cholerae clpV* mutant has a reduced T6SS-dependent killing activity toward *Escherichia coli*, suggesting that in this mutant sheath, contraction happens only once, and no subsequent toxin burst occurs (30). Disassembly of the *V. cholerae* VipA/VipB sheath (TssB/TssC homologs) (20, 21, 27) is dependent on a direct interaction between ClpV and the N-terminal helix of VipB (TssC homolog), which docks into a hydrophobic groove in the N-terminal domain of the ATPase (28).

*Pseudomonas aeruginosa* is an opportunistic pathogen, which has three T6SSs designated H1- to H3-T6SS (31, 32). Besides 13 conserved core genes, the *P. aeruginosa* H1-T6SS contains accessory genes, among them *tagJ*/*hsiE1*, which is not found in the H2- and H3-T6SSs. Although HsiE1 (also called TagJ) was found to interact with the essential sheath component HsiB1 (TssB/VipA homolog) (33), its exact role in T6SS remains unclear. Here we used a combination of structural and *in vivo* protein-protein interaction approaches to characterize molecular aspects of the H1-T6SS of *P. aeruginosa*. Structural characterization of the N domain of ClpV1 and analysis of its interaction with HsiC1 (TssC/VipB homolog) suggest that the H1-T6SS functions differently from the *V. cholerae* system. We solved the crystal structure of HsiE1 in complex with an N-terminal fragment of HsiB1 and observed that in addition to binding to HsiB1, HsiE1 is capable of interacting with ClpV1. We thus found evidence for distinct T6SS classes, which is confirmed through phylogenetic analysis of the four T6SS components, ClpV, HsiE/TagJ, TssB, and TssC.

## EXPERIMENTAL PROCEDURES

### 

#### 

##### Bacterial Strains, Plasmids, and Culture Conditions

Strains were cultivated in Luria-Bertani (LB) or Terrific broth at 37 °C. The antibiotics were added at the following concentrations: ampicillin, 100 μg/ml; kanamycin, 50 μg/ml. All bacterial strains and plasmids used are listed in Table 1.

**TABLE 1:** **Strains and plasmids used in this study**

	Relevant characteristics/Description	Source/Reference
**Strain (*E. coli*)**		
One-shotTOP10	F − mcrA Δ(mrr-hsdRMS-mcrBC) φ80lacZΔM15 ΔlacX74 recA1 araD139 Δ(araleu)	Invitrogen
DHM1	*cya-854 recA1 gyrA96* (NaI) *thi1 hsdR17 spoT1 rfbD1 glnV44*(AS)	Karimova (41)
B834(DE3)	F − *ompT hsdS*B (rB − mB − ) *gal dcm met* (DE3)	Laboratory collection

**Plasmid**		
pET28	Expression vector used for expression of N-terminal 6-histidine tagged proteins	Novagen
pET28-*hsiE1*	pET28 expressing HsiE1with an N-terminal histidine tag	This study
pET28-*hsiE1B1*	pET28 expressing HsiE1with an N-terminal histidine tag in tandem with untagged HsiB1	This study
pET28-*ClpV1-N*	pET28 expressing ClpV1-N with an N-terminal histidine tag	This study
pKT25	BTH vector for fusion of target proteins to *B. pertussis cya* gene T25 fragment; P_lac_::*cya*^1–675^p15ori, Km^R^	Karimova *et al.* (41)
pUT18C	BTH vector for fusion of target proteins to *B. pertussis cya* gene T18 fragment; P_lac_::*cya*^675–1197^pUCori, Ap^R^	Karimova *et al.* (41)
pKT25-*zip*	Fusion of *zip* encoding leucine zipper from GCN4 to *cya* gene T25 fragment in pKT25, Km^R^	Karimova *et al.* (41)
pUT18C-*zip*	Fusion of *zip* encoding leucine zipper from GCN4 to *cya* gene T18 fragment in pUT18C, Ap^R^	Karimova *et al.* (41)
pKT25-*hsiE1*	Fusion of *hsiE1* to *cya* gene T25 fragment in pKT25, Km^R^	Lossi *et al.* (33)
pUT18C-*hsiE1*	Fusion of *hsiE1* to *cya* gene T18 fragment in pUT18C, Ap^R^	Lossi *et al.* (33)
pKT25-*hsiB1*	Fusion of *hsiB1* to *cya* gene T25 fragment in pKT25, Km^R^	Lossi *et al.* (33)
pUT18C-*hsiB1*	Fusion of *hsiB1* to *cya* gene T18 fragment in pUT18C, Ap^R^	Lossi *et al.* (33)
pKT25-*hsiC1*	Fusion of *hsiC1* to *cya* gene T25 fragment in pKT25, Km^R^	Lossi *et al.* (33)
pUT18C-*hsiC1*	Fusion of *hsiC1* to *cya* gene T18 fragment in pUT18C, Ap^R^	Lossi *et al.* (33)
pKT25-*ClpV1*	Fusion of *ClpV1* to *cya* gene T25 fragment in pKT25, Km^R^	This study
pUT18C-*ClpV1*	Fusion of *ClpV1* to *cya* gene T18 fragment in pUT18C, Ap^R^	This study
pKT25-*N_ter_ClpV1*	Fusion of *N_ter_ClpV1* to *cya* gene T25 fragment in pKT25, Km^R^	This study
pUT18C-*N_ter_ClpV1*	Fusion of *N_ter_ClpV1* to *cya* gene T18 fragment in pUT18C, Ap^R^	This study
pKT25-*ClpV2*	Fusion of *ClpV2* to *cya* gene T25 fragment in pKT25, Km^R^	This study
pUT18C-*ClpV2*	Fusion of *ClpV2* to *cya* gene T18 fragment in pUT18C, Ap^R^	This study
pKT25-*N_ter_ClpV2*	Fusion of *N_ter_ClpV2* to *cya* gene T25 fragment in pKT25, Km^R^	This study
pUT18C-*N_ter_ClpV2*	Fusion of *N_ter_ClpV2* to *cya* gene T18 fragment in pUT18C, Ap^R^	This study
pKT25-*hsiE1*Δ*_1–10_*	Fusion of *hsiE1*Δ*_1–10_* to *cya* gene T25 fragment in pKT25, Km^R^	This study
pUT18C-*hsiE1*Δ*_1–10_*	Fusion of *hsiE1*Δ*_1–10_* to *cya* gene T18 fragment in pUT18C, Ap^R^	This study
pKT25-*hsiC1*Δ*_1–33_*	Fusion of *hsiC1*Δ*_1–33_* to *cya* gene T25 fragment in pKT25, Km^R^	This study
pUT18C-*hsiC1*Δ*_1–33_*	Fusion of *hsiC1*Δ*_1–33_* to *cya* gene T18 fragment in pUT18C, Ap^R^	This study
pKT25-*hsiC2*Δ*_1–30_*	Fusion of *hsiC2*Δ*_1–30_* to *cya* gene T25 fragment in pKT25, Km^R^	This study
pUT18C-*hsiC2*Δ*_1–30_*	Fusion of *hsiC2*Δ*_1–30_* to *cya* gene T18 fragment in pUT18C, Ap^R^	This study

##### Expression and Protein Purification

The *hsiE1* (*PA0086*) and *hsiB1* (*PA0083*) genes and the sequence corresponding to the first 159 residues of ClpV1 (*PA0090*) were amplified from *P. aeruginosa* PAO1 genomic DNA and cloned into pET28. pET28-E1 encodes HsiE1 with an N-terminal His tag cleavable with thrombin. pET28-E1B1 contained *hsiE1* preceded by sequence coding for a cleavable N-terminal His tag in frame with *hsiB1*. pET28-ClpV1-N encodes N-terminally His-tagged ClpV1-N. In all cases, transformed *E. coli* B834(DE3) cells were grown at 37 °C to an *A*_600_ of about 0.6 in Terrific broth. Expression of proteins was induced with 0.5 mm isopropyl 1-thio-β-d-galactopyranoside, and cells were grown overnight at 18 °C before centrifugation (4,000 × *g*, 15 min at 4 °C). Cell pellets were resuspended in buffer A (50 mm Tris-HCl, 500 mm NaCl, 20 mm imidazole (pH 8.0)) and lysed by French press after the addition of an anti-protease mixture (Sigma). Cell debris was eliminated by centrifugation (40,000 × *g*, 40 min).

Proteins were purified by IMAC chromatography using nickel-Sepharose resin (GE Healthcare) equilibrated in buffer A. Proteins were eluted with buffer A containing 500 mm instead of 20 mm imidazole and were further purified by size exclusion chromatography using a HiLoad Superdex 75 column equilibrated in 50 mm Tris-HCl and 250 mm NaCl (pH 8). All chromatographic steps were performed on an ÄKTAprime Plus system (GE Healthcare) at 4 °C. Protein purity was checked by SDS-PAGE. Proteins were concentrated to at least 10 mg/ml using centrifugal concentrators (Millipore) and stored at −80 °C.

##### Crystallization and Structure Determination

HsiE1 (at 15 mg/ml) crystallized in 30% 2-methyl 2,4-pentanediol, 0.1 m sodium acetate (pH 4.6), and 20 mm CaCl_2_. For co-crystallization, HsiB1 peptide (MGSTTSSQKFIARNRAPRVQ; Eurogenetec) was added in a 5× molar excess to HsiE1 concentrated to 22 mg/ml. Crystals grew in 50 mm MES (pH 6.5), 100 mm ammonium acetate, 10% glycerol, and 28% PEG 8000. Crystals of HsiE1-HsiB1 fragment grew in 100 mm Tris (pH 8.0), 24% PEG 6000, and 0.2 m CaCl_2_. ClpV crystals grew in 0.2 m sodium malonate and 20% PEG 3350. Crystals that were not already cryoprotected by the crystallization solution were transferred into buffer containing 25% glycerol.

Data were collected on a MicroMax-007 HF rotating anode (Rigaku) and at Diamond Light Source beamlines I03, I04, and I04-1 and reduced in XDS (34) and iMosflm (35). Structures were solved in Phaser (36). VPA1052 (37) was used as a search model to solve HsiE1, which was in turn used to solve the structure of the HsiE1-HsiB1 complexes. ClpV-N (28) was used as a search model for the ClpV1-N domain. Structures were refined in Refmac5 (38) and phenix.refine (39) and rebuilt in *Coot* (40) until convergence. Crystallographic statistics are summarized in Table 2. All models and structure factors were deposited to the Protein Data Bank with codes 4UQW (ClpV N domain), 4UQX (HsiE1), 4UQY (HsiE1 + HsiB1 peptide), and 4UQZ (HsiE1 + HsiB1 fragment).

**TABLE 2:** **Data collection and refinement statistics**

	HsiE1	HsiE1 + HsiB1 peptide	HsiE1 + HsiB1 fragment	ClpV N
**Data collection**				
Space group	P 21	P 21 21 21	P 21 21 21	P 41

**Cell dimensions**				
*a*, *b*, *c* (Å)	56.8, 45.9, 59.5	45.2, 64.0, 94.5	52.1, 67.3, 94.0	90.5, 90.5, 54.3
α, β, γ (degrees)	90, 110.0, 90	90, 90, 90	90, 90, 90	90, 90, 90
Resolution (Å)	55.9–1.2 (1.22–1.20)	53.0–1.6 (1.63–1.60)	54.7–1.6 (1.63–1.60)	90.5–1.5 (1.53–1.50)
*R*_merge_	0.055 (0.527)	0.061 (0.59)	0.084 (0.442)	0.08 (0.71)
*I*/σ(*I)*	8.2 (1.9)	10.7 (2.2)	11.4 (2.7)	8.8 (1.9)
Completeness (%)	98.1 (98.2)	99.8 (98.5)	98.1 (96.0)	99.9 (99.9)
Redundancy	2.7 (2.7)	3.2 (3.2)	5.0 (4.1)	4.6 (4.7)

**Refinement**				
No. of reflections	88,118 (4,483)	36,309 (1,772)	43,478 (2,088)	70,465 (3,476)
*R*_work_ / *R*_free_	0.151 / 0.158	0.177 / 0.205	0.188 / 0.221	0.159 / 0.181

**No. of atoms**				
Protein	2237	2135	2191	2564
Ligand/ion	20	-	4	9
Water	324	197	314	358

***B*-Factors**				
Protein	14.2	29.0	25.1	26.6
Ligand/ion	23.9	-	37.6	25.7
Water	28.9	37.5	35.7	40.2

**r.m.s. deviations**				
Bond lengths (Å)	0.008	0.006	0.012	0.016
Bond angles (degrees)	1.29	1.03	1.41	1.65
PDB code	4UQX	4UQY	4UQZ	4UQWx

*^a^* Values in parentheses are for the highest resolution shell.

##### Bacterial Two-hybrid Assay

The genes of interest were amplified from *P. aeruginosa* PAO1 genomic DNA, adding appropriate restriction sites. The resulting PCR products were ligated into either bacterial two-hybrid (BTH) plasmid pKT25 or pUT18C, leading to in-frame fusions of the protein of interest with the T25 or T18 subunit of the *Bordetella pertussis* adenylate cyclase, respectively (41). Recombinant pKT25 and pUT18C plasmids were co-transformed into the reporter *E. coli* DHM1 strain. Four independent colonies for each co-transformation were inoculated into LB medium supplemented with ampicillin and kanamycin. After overnight growth at 37 °C, 10 μl of each culture were spotted onto MacConkey agar plates with 1% maltose and LB agar plates supplemented with 5-bromo-4-chloro-3-indolyl β-d-galactoside (X-gal), both in the presence of ampicillin, kanamycin, and 1 mm isopropyl 1-thio-β-d-galactopyranoside, and incubated for at least 48 h at 30 °C. The pKT25 and pUT18C derivatives encoding the leucine zipper from GCN4, which readily dimerizes, were used as a positive control in all experiments. The experiments were done at least in duplicate, and a representative result is shown.

For quantification of BTH interactions, β-galactosidase activity from co-transformants picked from X-gal LB agar plates was measured as described previously (42). The β-galactosidase activity is calculated in Miller units.

##### Bioinformatics Analysis

For analysis of the groove residues, protein sequences were retrieved by BlastP searches using each of ClpV, ClpV1, ClpV2, and ClpV3 as queries. After pruning of duplicates, a total of 1,593 sequences were aligned with MAFFT (43). For the phylogenetic analysis, sequences from 68 T6SSs were retrieved from the Kyoto Encyclopedia of Genes and Genomes. Strains and accession codes are shown in Table 3. Sequences were aligned with MAFFT. For ClpV and TssC, the e-ins-i option (multiple conserved domains and long gaps) was used. TssB and TagJ/HsiE were aligned with default parameters. In all four cases, the Blosum62 scoring matrix was used. Maximum likelihood phylogenies were calculated with phyML (44) with the LG substitution model, no invariable sites, nearest neighbor interchange tree improvement, topology and branch optimization, and aBayes branch support calculation. Trees were visualized with TreeDyn (45). The weblogos were created at WebLogo 3 Web site.

**TABLE 3:** **List of strains used in the phylogenetic analysis**

Organism	Code	HsiE1/TagJ	HsiB/TssB	HsiC/TssC	ClpV
*Acidovorax citrulli* AAC00–1	aav	Aave_1478	Aave_1476	Aave_1477	Aave_1482
*Achromobacter xylosoxidans* A8	axy	AXYL_06394	AXYL_06397	AXYL_06396	AXYL_06389
		None	AXYL_05693	AXYL_05692	AXYL_05687
*Aeromonas hydrophila* ATCC 7966	aha	None	AHA_1832	AHA_1833	AHA_1841
*Agrobacterium tumefaciens* C58	atu	Atu4339	Atu4342	Atu4341	Atu4344
*Azoarcus sp*. BH72	azo	azo3899	azo3895	azo3896	azo3903
*Bordetella bronchiseptica* RB50	bbr	BB0803	BB0800	BB0801	BB0810
*Bradyrhizobium japonicum* USDA 6	bju	None	BJ6T_33630	BJ6T_33620	BJ6T_33570
*Burkholderia cenocepacia* J2315	bcj	None	BCAL0341	BCAL0342	BCAL0347
*Burkholderia thailandensis* E264	bte	BTH_II0138	BTH_II0121	BTH_II0122	BTH_II0140
		None	BTH_I2964	BTH_I2963	BTH_I2958
		None	BTH_II1901	BTH_II1900	BTH_II1895
		None	BTH_II0870	BTH_II0869	BTH_II0864
		None	BTH_II0258	BTH_II0259	BTH_II0264
*Cronobacter sakazakii* ES15	csk	ES15_3835	ES15_3846	ES15_3845	ES15_3830
		ES15_2806	ES15_2819	ES15_2818	ES15_2825
*Cupriavidus taiwanensis* LMG 19424	cti	None	RALTA_A0608	RALTA_A0609	RALTA_A0607
		None	RALTA_B1013	RALTA_B1014	RALTA_B1019
*Dechloromonas aromatica* RCB	dar	None	Daro_2177	Daro_2176	Daro_2171
*Delftia acidovorans* SPH-1	del	Daci_3856	Daci_3850	Daci_3851	Daci_3864
*Escherichia coli* O6:K2:H1 CFT073	ecc	None	c3385	c3386	c3392
*Escherichia coli* O157:H7 Sakai (EHEC)	ecs	None	ECs0233	ECs0231	ECs0223
*Edwardsiella piscicida* C07-087	etc	None	ETAC_09445	ETAC_09440	ETAC_09415
*Francisella tularensis* TIGB03	ftg	None	FTU_1718	FTU_1717	FTU_1770
*Klebsiella pneumoniae* 342	kpe	None	KPK_3069	KPK_3068	KPK_3063
*Leptothrix cholodnii* SP-6	lch	None	Lcho_4091	Lcho_4090	Lcho_4084
*Mesorhizobium loti* MAFF303099	mlo	None	mlr2337	mlr2338	mll2335
*Methylomicrobium alcaliphilum*	mah	MEALZ_1934	MEALZ_1931	MEALZ_1932	MEALZ_1938
*Myxococcus xanthus* DK 1622	mxa	None	MXAN_4807	MXAN_4808	MXAN_4813
*Paracoccus aminophilus* JCM 7686	pami	None	JCM7686_pAMI6p160	JCM7686_pAMI6p159	JCM7686_pAMI6p154
*Paracoccus denitrificans* PD1222	pde	None	Pden_2443	Pden_2444	Pden_2440
*Pantoea ananatis* LMG 20103	pam	PANA_2358	PANA_2366	PANA_2365	PANA_2354
		None	PANA_4151	PANA_4150	PANA_4145
*Pectobacterium atrosepticum* SCRI1043	eca	None	ECA3445	ECA3444	ECA3436
*Pelobacter carbinolicus* DSM 2380	pca	None	Pcar_2814	Pcar_2815	Pcar_2820
*Photorhabdus luminescens* TTO1	plu	None	plu2301	plu2300	plu2287
		None	plu0372	plu0371	plu0363
*Pseudomonas putida* F1	ppf	None	Pput_2622	Pput_2623	Pput_2627
*Pseudomonas syringae* B728a	psb	None	Psyr_4953	Psyr_4954	Psyr_4958
*Pseudomonas aeruginosa* PA01	pae	PA0086	PA0083	PA0084	PA0090
		None	PA1657	PA1658	PA1662
		None	PA2365	PA2366	PA2371
*Pseudomonas fluorescens* SBW25	pfs	None	PFLU6019	PFLU6020	PFLU6025
*Rahnella aquatilis* HX2	raa	None	Q7S_25121	Q7S_25126	Q7S_25191
		None	Q7S_23336	Q7S_23341	Q7S_23366
*Ralstonia eutropha* JMP134	reu	None	Reut_A1733	Reut_A1732	Reut_A1727
		None	Reut_B5266	Reut_B5265	Reut_B5260
*Ralstonia solanacearum* GMI1000	rso	None	RS01965	RS01964	RS01959
*Rhizobium leguminosarum* 3841	rle	pRL120471	pRL120474	pRL120473	pRL120476
*Rhodobacter sphaeroides* 2.4.1	rsp	None	RSP_3477	RSP_3478	RSP_3474
*Salmonella typhimurium* LT2	stm	STM0270	STM0273	STM0274	STM0272
*Serratia marcescens* WW4	smw	SMWW4_v1c30250	SMWW4_v1c30140	SMWW4_v1c30150	SMWW4_v1c30290
		None	SMWW4_v1c31600	SMWW4_v1c31590	SMWW4_v1c31540
*Solibacter usitatus* Ellin6076	sus	Acid_0234	Acid_0231	Acid_0232	Acid_0239
*Taylorella equigenitalis* MCE9	teq	TEQUI_0721	TEQUI_0718	TEQUI_0719	TEQUI_0708
*Variovorax paradoxus* S110	vap	Vapar_0196	Vapar_0193	Vapar_0194	Vapar_0200
		Vapar_0525	Vapar_0550	Vapar_0549	Vapar_0530
*Vibrio cholerae* O1 biovar El Tor N16961	vch	None	VCA0107	VCA0108	VCA0116
*Vibrio parahaemolyticus* RIMD 2210633	vpa	VPA1032	VPA1035	VPA1034	VPA1028
		None	VP1402	VP1403	VP1392
*Xanthomonas oryzae* KACC 10331	xoo	None	XOO3481	XOO3480	XOO3475
		XOO3049	XOO3052	XOO3051	XOO3045
*Xenorhabdus bovienii* SS-2004	xbo	None	XBJ1_2103	XBJ1_2102	XBJ1_2096
		None	XBJ1_0263	XBJ1_0264	XBJ1_0272
*Yersinia pestis* KIM10	ypk	y1560	y1545	y1546	y1538
		None	y2706	y2705	y2699
		None	y3369	y3368	y3362
		None	y3675	y3674	y3669

## RESULTS

### 

#### 

##### Distinct Structural Features of the N-terminal Domain of P. aeruginosa ClpV1

**FIGURE 1.**
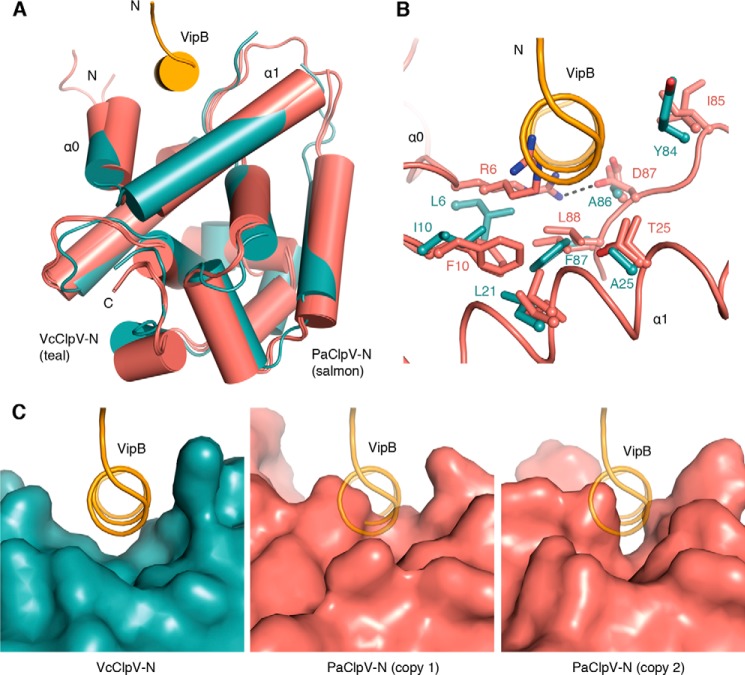
**Structural differences between *P. aeruginosa* ClpV1 and *V. cholerae* ClpV.** *A*, superposition of two copies of ClpV1-N (*salmon*) onto *V. cholerae* ClpV-N (*teal*) (Protein Data Bank code 3ZRJ) shows overall structural conservation. The peptide mimicking the N terminus of VipB that binds to a groove formed by helices α0 and α1 of ClpV-N is shown as an *orange cylinder*. The N and C termini of ClpV-N are indicated. *B*, *close-up view* of the superposed grooves between helices α0 and α1 in ClpV1-N (*salmon*) and *V. cholerae* ClpV-N (*teal*). The view is rotated clockwise by 20° with respect to *A*. Residues facilitating binding of VipB in ClpV-N and their counterparts in both copies of ClpV1-N are shown as *sticks*. In either ClpV1-N copy, two bulky charged residues, Arg^6^ and Asp^87^, obstruct the groove. *C*, surface representations of ClpV-N (*left*) and the two copies of ClpV1-N (*middle* and *right*) show that the binding groove is blocked in *P. aeruginosa*. The view is as in *B*.

The structure of the N-terminal domain of *V. cholerae* ClpV (ClpV-N) has previously been solved in complex with a peptide corresponding to the N-terminal helix of the TssC homolog VipB (Table 4) (28). A hydrophobic groove in ClpV-N provides the binding site for VipB. To investigate whether the N-terminal domain of *P. aeruginosa* ClpV1 (ClpV1-N) can interact with the TssC homolog HsiC1 in a similar manner, we solved the structure of ClpV1-N from crystals diffracting to 1.5 Å, using the *Vibrio* structure (Protein Data Bank code 3ZRJ) as a model for molecular replacement (Table 2). The asymmetric unit contains two copies of ClpV1-N in nearly identical conformations (r.m.s. deviation for all Cα atoms of 0.5 Å). The overall structure of ClpV1-N is similar to that of the *Vibrio* ClpV-N, with matching secondary structure elements and an r.m.s. deviation of 1.6 Å for 121 equivalent Cα atoms (Fig. 1*A*). Both structures possess an N-terminal helix α0 that distinguishes ClpV-type ATPases from other Hsp100 ATPases (*e.g.* ClpA) (46). A hydrophobic cleft between this helix and helix α1 forms the binding site for VipB in *V. cholerae* (Fig. 1*A*).

**TABLE 4:** **Names of T6SS components used in this study** Given are protein names in the three T6SSs in *P. aeruginosa* and in *V. cholerae*. Tss, type six secretion.

Generic name	*P. aeruginosa*	*V. cholerae*	Function
TssB	HsiB1,2,3	VipA	Sheath component
TssC	HsiC1,2,3	VipB	Sheath component
TagJ	HsiE1		Unknown
ClpV	ClpV1,2,3	ClpV	Sheath disassembly

The residues lining the VipB binding groove in ClpV-N are partially conserved in ClpV1-N. For example, Ile^10^ and Phe^87^ lie at the bottom of the groove. The corresponding residues in ClpV1-N are Phe^10^ and Leu^88^ (Fig. 1*B*). However, other residues are not conserved. Tyr^84^, which packs against Glu^22^ of VipB in *V. cholerae*, is Ile^85^ in *P. aeruginosa*, a residue that could not participate in the same interaction. The two aliphatic residues Leu^6^ and Ala^86^, which sit at the top of the groove in *Vibrio* ClpV-N, are replaced by large residues of opposite charge in ClpV1-N (*i.e.* Arg^6^ and Asp^87^) (Fig. 1*B*). In one of the two ClpV1-N molecules in the asymmetric unit, these residues form a salt bridge across the groove, whereas in the other, they point into the groove. Both conformations not only diminish the hydrophobic character of the groove but also are incompatible with binding of an α-helix, as observed in *V. cholerae* (Fig. 1, *B* and *C*). Consistent with the altered conformation of the binding groove, we were unable to obtain a complex of ClpV1-N bound to a peptide corresponding to the N terminus of HsiC1.

##### Phylogenetic Analysis Identifies Distinct T6SS Classes Based on the ClpV Structure

**FIGURE 2.**
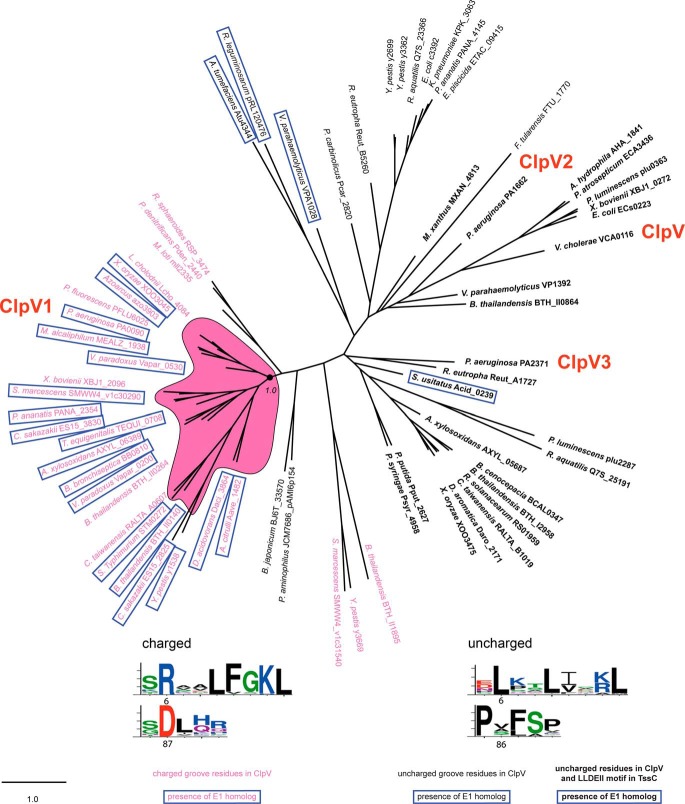
**Division of T6SSs into distinct phylogenetic classes.** Maximum likelihood phylogenetic tree generated from 68 aligned ClpV sequences belonging to the indicated bacterial species. ClpV sequences separate into two classes according to two residues in the hydrophobic groove between helices α0 and α1. Homologs where these residues are uncharged like in *V. cholerae* are shown in *black*, whereas homologs with charged residues are shown in *pink. Blue boxes* indicate the presence of an HsiE1 homolog on the same secretion cluster. *Boldface type* denotes the presence of the LLDEII motif in the TssC homolog on the same cluster. The branch that is highly enriched in T6SSs containing ClpV homologs with charged residues and HsiE1 homologs is shown against a *pink background*. The branch support value is shown. The three ClpV homologs from *P. aeruginosa* and *V. cholerae* ClpV are indicated in *red*. The *weblogos* at the *bottom* show the sequence conservation of residues in the hydrophobic groove of ClpV, depending on the presence (*left*) or absence (*right*) of the pair of charged residues. On the *left*, the two charged residues are *numbered* according to the *P. aeruginosa* ClpV1 sequence as in our structure. On the *right*, the uncharged residues in the same positions are *numbered* according to the *V. cholerae* ClpV sequence, as in the published structure (28). The *scale bar* shows amino acid changes per site. At the *bottom* is a brief *key* to the labeling used.

Alignment of ClpV sequences shows a division based on sequence conservation within the binding groove in the N-terminal domain (Fig. 2, *weblogos*). There are homologs with an arginine and an aspartate (rarely glutamate) as in *P. aeruginosa* ClpV1 (Arg^6^ and Asp^87^, respectively) and homologs with two aliphatic residues as in *V. cholerae* ClpV (Leu^6^ and Ala^86^, respectively). Phylogenetic analysis of ClpV sequences from 68 T6SSs covering a wide range of bacterial species shows evolutionary separation based on the groove residues. ClpV homologs with charged residues group with *P. aeruginosa* ClpV1 on the *left side* of the phylogenetic tree (Fig. 2, shown in *pink*). ClpV homologs with aliphatic residues group with *V. cholerae* ClpV on the *right side* of the tree (Fig. 2, shown in *black*). It is noticeable that the two other *P. aeruginosa* ClpVs, ClpV2 and ClpV3, which are associated with the H2- and H3-T6SSs, respectively, lack the charged residues found in the hydrophobic groove of ClpV1 and group with *V. cholerae* ClpV (Fig. 2, shown in *black*).

##### TssC Has Coevolved with ClpV

**FIGURE 3.**
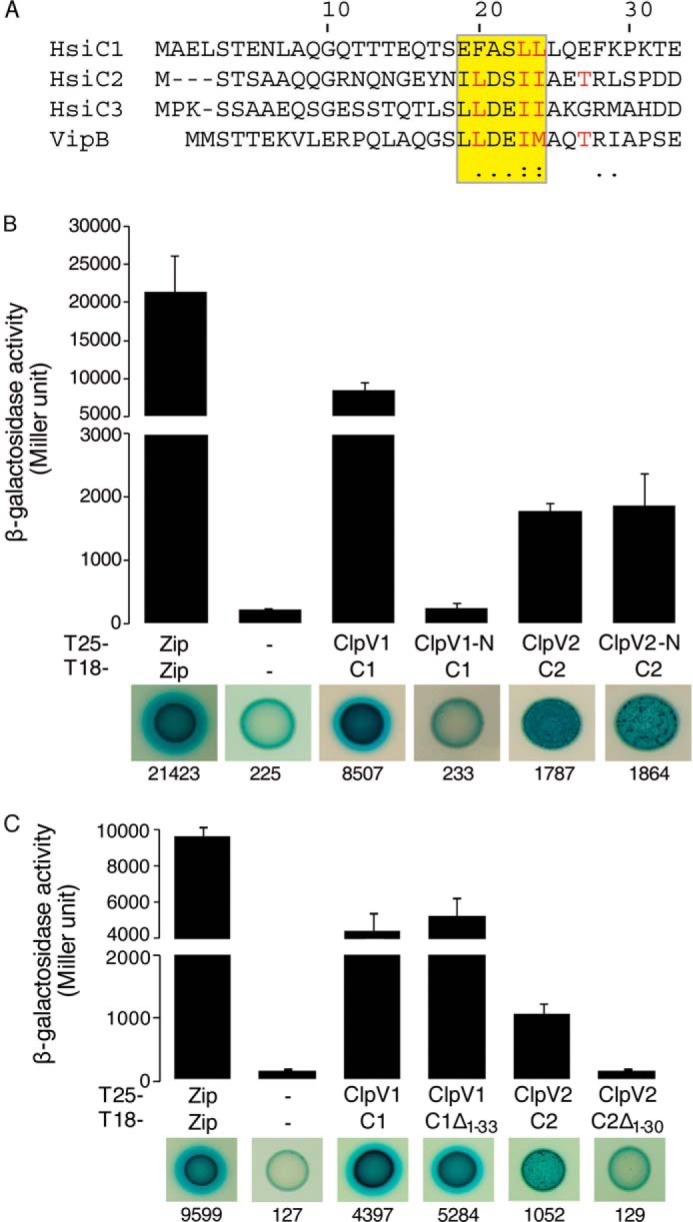
**Role of the N-terminal α-helices of HsiC proteins in the interaction with their cognate ClpV.** *A*, sequence alignment of the N terminus (residues 1–33) of the *P. aeruginosa* proteins HsiC1, HsiC2, and HsiC3 and of VipB, the HsiC homolog of *V. cholerae*. The conserved binding motif is *highlighted*. Residues implicated in binding in the ClpV-VipB complex are shown in *red*. Degrees of conservation are indicated by *asterisks*, *colons*, and *dots below* the alignment. *B*, BTH assays analyzing the interaction between the N-terminal domain of ClpV1 and full-length HsiC1 and between the N-terminal domain of ClpV2 and full-length HsiC2. A graphical representation of the β-galactosidase activity from co-transformants is shown (*top*), and images of corresponding spots on X-gal LB agar plates are displayed (*bottom*). *Zip*, leucine zipper domain of the yeast transcription factor GCN4. T6SS proteins are HsiC1 (*C1*), HsiC2 (*C2*), N-terminal domain of ClpV1 (*ClpV1-N*), and N-terminal domain of ClpV2 (*ClpV2-N*). *C*, ability of truncated forms of HsiC1 and HsiC2 to interact with their cognate ClpV as determined by BTH assays. The combinations of T25/T18 fusion proteins are indicated, and abbreviations are used as described above. Additionally, *C1*Δ_*1–33*_ represents HsiC1 truncated for the first 33 residues (including those forming the first two predicted helices), and *C1*Δ_*1–30*_ indicates HsiC2 truncated for the first 30 residues. Experiments were carried out in duplicate. *Error bars*, S.E.

In *V. cholerae*, the peptide corresponding to the N-terminal α-helix of VipB that binds to ClpV contains a short conserved motif (^19^LLDEIM^24^) (28). By analyzing the N termini of the *P. aeruginosa* VipB homologs, HsiC1, HsiC2, and HsiC3, we found this motif to be conserved in C2 (^18^ILDSII^23^) and C3 (^20^LLDEII^25^) but not C1 (^21^EFASLL^26^) (Fig. 3*A*). Mapping the presence of the LLDEII motif in TssC proteins onto the phylogenetic tree of ClpV sequences suggests that TssC homologs have coevolved with ClpV ATPases (Fig. 2, *boldface type*). TssC homologs with the LLDEII motif are associated only with ClpV homologs having aliphatic residues in their hydrophobic groove.

To investigate these differences and the interaction between the ClpV and TssC partners, we used the BTH assay. Although full-length ClpV1 interacts with HsiC1, the isolated N-terminal domain does not (Fig. 3*B*), in contrast to what was observed in *V. cholerae* (28). The two other *P. aeruginosa* ClpV proteins display binding grooves that are closely related to the one observed in *Vibrio* ClpV-N (Phyre homology models; data not shown). In contrast to ClpV1 and HsiC1, the N-terminal domain of ClpV2 interacts with HsiC2 to the same degree as full-length ClpV2 (Fig. 3*B*), suggesting that the interaction mode between ClpV2 and HsiC2 is similar to the *Vibrio* ClpV-VipB interaction.

We then engineered an N-terminal truncation of HsiC1 to remove the first 33 residues (HsiC1Δ_1–33_). HsiC1Δ_1–33_ interacts with ClpV1 (Fig. 3*C*), which suggests that the N terminus of HsiC1 is not essential for a stable interaction, further highlighting the difference with *V. cholerae*. In contrast, the truncation of the corresponding N-terminal residues of HsiC2 (HsiC2Δ_1–30_) has a detrimental effect (10-fold reduction) on binding to ClpV2 (Fig. 3*C*). These data provide evidence for at least two distinct classes of TssC homologs that specifically interact with cognate ClpVs, which is consistent with the phylogenetic analysis. One mode of interaction, exemplified by *V. cholerae*, involves a primary interaction between the ClpV N domain and the N-terminal helix of TssC (VipB/HsiC) within a hydrophobic groove. The other mode involves additional features beyond the N domain of ClpV or additional protein partners, which may modulate the TssC-ClpV interaction.

##### HsiE1/TagJ Is a Direct T6SS Partner for ClpV1

**FIGURE 4.**
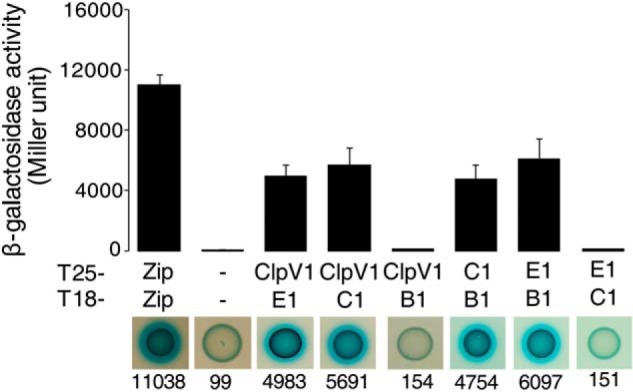
**BTH assays showing that ClpV1 interacts with HsiE1 and HsiC1.** In the *top panel*, graphical representations of β-galactosidase activity from co-transformants of *E. coli* DHM1 cells expressing the indicated proteins fused to the adenylate cyclase T25 or T18 subunit are shown. Images of the corresponding bacterial spots on X-gal LB agar plates are shown in the *bottom panel* with the corresponding average activity in Miller units indicated *below* each image. The combinations of T25/T18 fusion proteins are indicated and abbreviated as described in the legend to Fig. 3. *B1*, HsiB1; *E1*, HsiE1. Experiments were carried out in triplicate. *Error bars*, S.E.

T6SS clusters vary in genetic organization and are composed of core and accessory genes. The *hsiE1*/*tagJ* gene is found in the *P. aeruginosa* H1-T6SS cluster but is lacking in the H2- and H3-T6SSs or in the *V. cholerae* T6SS (Table 4). We performed BTH experiments showing that HsiE1 interacts with full-length ClpV1 (Fig. 4). The interaction between ClpV1 and HsiE1 distinguishes *P. aeruginosa* from *V. cholerae* where no HsiE/TagJ homolog exists. Analysis of the ClpV phylogenetic tree further showed that when the T6SS involves a TssC protein containing the LLDEII motif, such as in *V. cholerae*, there is no associated HsiE1 homolog (Fig. 2, *boldface type* and *not boxed*). There is only one exception in *Solibacter usitatus*. This suggests that these two features are mutually exclusive and that HsiE1 plays a role in the interaction between ClpV and the TssC/TssB sheath.

##### Crystal Structure of HsiE1 in Complex with the N Terminus of HsiB1

**FIGURE 5.**
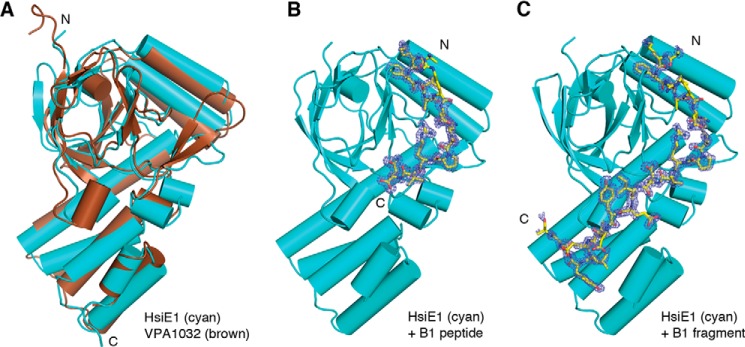
**Structure of HsiE1.** *A*, *P. aeruginosa* HsiE1 (*cyan*) is structurally similar to VPA1032, the HsiE1 homolog from *V. parahemeolyticus* (*brown*). The N and C termini of both proteins are indicated. *B*, a peptide corresponding to the N terminus of HsiB1 (*yellow sticks*) binds to HsiE1 (*cyan*). An *F_o_* − *F_c_* omit map contoured at 3σ is shown as *blue mesh around* the HsiB1 peptide. The N and C termini of the HsiB1 peptide are indicated. *C*, an N-terminal fragment of HsiB1 (*yellow sticks*) binds to HsiE1 (*cyan*). The *panel* is designed like *B*. All three panels display HsiE1 in the same orientation.

We previously showed that *P. aeruginosa* HsiE1 interacts with the TssB sheath component HsiB1 (Fig. 4) (33). Here, we solved the HsiE1 structure from crystals diffracting to 1.2 Å (Table 2). *Vibrio parahemeolyticus* VPA1032, a protein of unknown function (37) with 26% sequence identity to HsiE1 (Protein Data Bank code 1ZBP), served as a model for molecular replacement. Both VPA1032 and HsiE1 belong to the “ImpE family” (47). Their structures are similar, as indicated by the r.m.s. deviation of 1.5 Å over 199 equivalent Cα atoms and the conserved N-terminal tetratricopeptide repeat domain and C-terminal “ImpE fold” (Fig. 5*A*).

**FIGURE 6.**
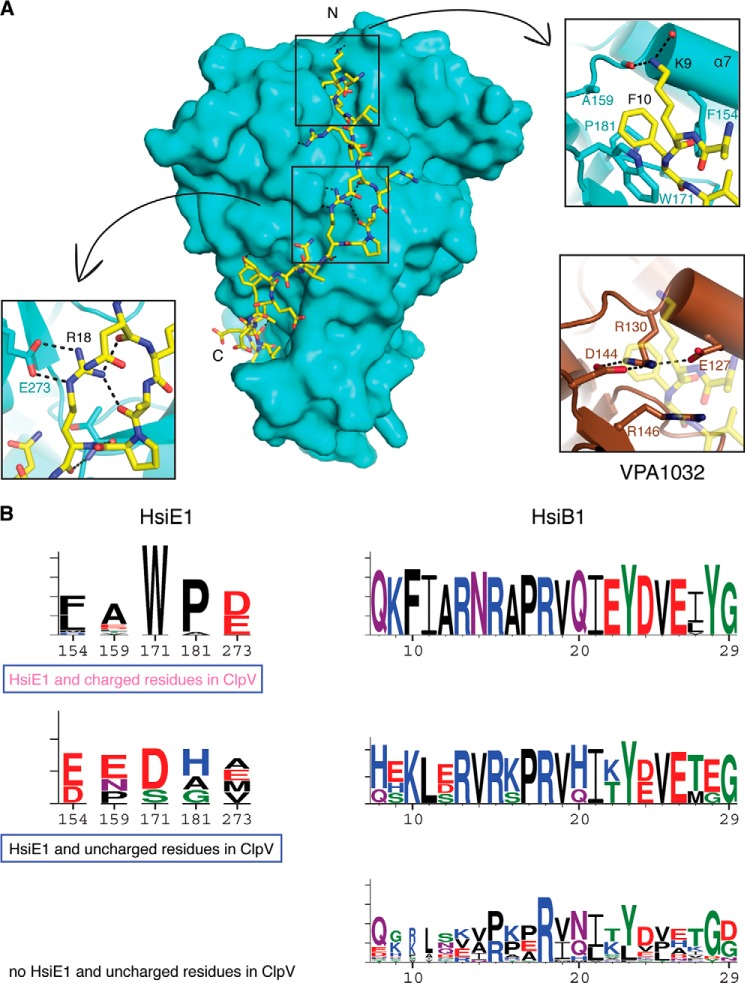
**Interaction between HsiE1 and HsiB1.** *A*, the HsiB1 fragment binds in a groove on the surface of HsiE1 (*main panel*). The N and C termini of the HsiB1 fragment are indicated. Two regions of interaction are *boxed* and *magnified* (*arrows*). The *top right inset* shows the pocket on HsiE1 into which HsiB1 Tyr^12^ binds and the interaction between HsiB1 Lys^9^ and helix α7 in HsiE1. The *bottom left inset* shows the salt bridges between HsiB1 Arg^18^ and HsiE1 Glu^273^. The *bottom right inset* shows the region in VPA1032 corresponding to the HsiB1 binding pocket in HsiE1. HsiB1 is shown in *pale yellow. B*, *weblogos* derived from sequence alignments of HsiE1 (*left*) and HsiB1 (*right*) homologs. For HsiE1, residues are shown that interact with HsiB1 in our structure. For HsiB1, the residues that are visible in our structure are shown. Residue numbers are indicated and match those in *A*. The *top panels* show the sequence conservation in T6SSs containing a ClpV homolog with charged residues and an HsiE1 homolog. The *middle panels* show the sequence conservation in T6SSs containing a ClpV homolog with uncharged residues and an HsiE1 homolog. This matches the *bottom right inset* in *A*. The *bottom panel* shows the sequence conservation of TssB from T6SSs containing a ClpV homolog with uncharged residues but no HsiE1 homolog. All three sets of *panels* carry a *label* that is also found in the phylogenetic tree in Fig. 2.

We crystallized HsiE1 in complex with a peptide corresponding to the first 20 residues of HsiB1 (^1^MGSTTSSQKFIARNRAPRVQ^20^) or an HsiB1 degradation product, which appeared during purification of the E1-B1 complex. Both the E1-B1 peptide and E1-B1 fragment structures were solved by molecular replacement (Table 2 and Fig. 5, *B* and *C*). In the E1-B1 complex structures, there is clear electron density for residues 8–20 of the extraneously added B1 peptide (Fig. 5*B*) and for residues 8–30 of the co-purified B1 fragment (Fig. 5*C*), respectively. Both structures are nearly identical to the structure of HsiE1 alone, with an r.m.s. deviation of 0.4 Å in either case, and to each other with an r.m.s. deviation of 0.3 Å. The B1 fragment (residues 8–30) wraps around E1 in an extended conformation (Fig. 6*A*, *main panel*), following a prominent surface groove that originates in a deep hydrophobic pocket between the 12-stranded β-barrel of the “ImpE fold” and the two α-helices just preceding it (Fig. 6*A*, *left inset*). The B1 residue Phe^10^ nestles in that pocket, which is lined with residues Phe^154^, Trp^171^, and Pro^181^.

The amino group of Lys^9^ of HsiB1 interacts with the negative dipole of helix α7 and forms hydrogen bonds with two carbonyl oxygens at the end of that helix (residues 155 and 157). Another key residue for the interaction is Arg^18^ of B1, which forms two salt bridges with the carboxyl group of Glu^273^ in HsiE1 (Fig. 6*A*, *top right inset*). In addition, most of the E1-B1 binding interface is hydrophobic in nature, involving Ala^12^, Gln^20^, Tyr^23^, Val^25^, and Leu^27^ from B1.

The residues of B1 between 8 and 29 that are involved in specific E1 interactions are highly conserved (Fig. 6*B*, *top*). HsiE1 homologs also show conservation of residues that line the hydrophobic pocket with residues 171 and 181 always tryptophan and proline, respectively, and residue 154 always a large hydrophobic residue (*e.g.* Phe, Leu, or Trp). Residue 273, which forms two salt bridges with the conserved Arg^18^ in HsiB1, is always glutamic or aspartic acid.

##### The N Terminus of TssB Homologs Coevolved with HsiE1 Homologs

Because we showed that *P. aeruginosa* HsiE1 interacts both with ClpV1 and HsiB1, it is tempting to generalize that once HsiE1 binds onto the sheath via an interaction with TssB, it is an ideal docking point for ClpV1 to be recruited to the sheath. There are features in the N termini of TssB proteins that support this idea. The conservation of the N termini of TssB homologs associated with HsiE1 has been described above (Fig. 6*B*, *top*). The N termini of TssB homologs not associated with HsiE1 do not show this striking conservation (Fig. 6*B*, *bottom*), and there is very little similarity between the two sets. Features in the N termini of TssB homologs, therefore, also appear to differentiate between the two T6SS classes.

## DISCUSSION

Repeated injection of T6SS-dependent toxins is essential for effective killing of target cells, and rebuilding of the secretion sheath is necessary in this process. Here, we present molecular details of T6SS components involved in the sheath dynamics and propose the existence of distinct T6SS classes based on their interaction and co-evolution. It has been previously shown that the formation of the contractile sheath of the T6SS requires the co-polymerization of TssB/HsiB1/VipA and TssC/HsiC1/VipB components (Table 4) (20, 21, 23, 24). For sheath disassembly, the AAA^+^ ATPase ClpV is required (21, 30). In *V. cholerae*, the ClpV N-domain directly interacts with the VipB component (TssC/HsiC1 homolog) of the sheath via a conserved hydrophobic groove and the N-terminal helix of VipB (28). We identified two key residues that have diverged from their equivalents in *V. cholerae* in a subset of T6SSs. These residues are of complementary charge and alter the shape and accessibility of the ClpV1-N groove, as shown in the structure of the N-terminal domain of ClpV1 from *P. aeruginosa* that we solved. The presence of these residues is incompatible with the mode of binding observed for ClpV-VipB. Consequently, we found that the N domain of the *Pseudomonas* ATPase ClpV1 is not sufficient for interaction with HsiC1, nor is the presence of the N-terminal helix of HsiC1 required for it. This is markedly different from what was observed in *V. cholerae*, where VipB binds to ClpV via its N-terminal helix containing a LLDEII motif (28). The differences in the interaction between ClpV and the sheath component TssC in the H1-T6SS of *P. aeruginosa versus V. cholerae* lead us to hypothesize that sheath disassembly differs mechanistically in these systems.

To provide further evidence for this hypothesis, we carried out phylogenetic analysis of T6SSs and mapped our structural and interaction data on the resulting tree. Our phylogenetic tree of ClpV divides into two main branches based on the presence of residues of opposite charge on top of the hydrophobic binding groove of the N domain. We found that TssC sequences divide similarly according to the presence of the N-terminal LLDEII motif, which is not observed in secretion systems where the ClpV homolog contains charged residues in the hydrophobic groove. On these features we could superimpose the observation that the accessory protein TagJ/HsiE co-occurs only with the *Pseudomonas* ClpV1 type and thus co-evolved with TssC homologs that lack the LLDEII motif.

The importance of the co-evolution between TagJ/HsiE and the two classes of ClpV and TssC is reinforced by two observations. First, we were able to show that HsiE1 directly interacts with ClpV1, which suggests that the function of these two proteins is coordinated. Second, we have shown previously that HsiE1 directly interact with the other sheath component HsiB1/TssB, suggesting that ClpV1 and HsiE1 function is associated with sheath disassembly. Here, we have solved the structure of HsiE1 in complex with residues 8–30 of the *P. aeruginosa* HsiB1. The N terminus of HsiB1 wraps around HsiE1 in an extended conformation, and all HsiB1 residues visible in the crystal structure are in close proximity to HsiE1. Residues 8–29 are highly conserved in HsiB1 homologs (TssB) from secretion systems containing HsiE1. It is thus clear from our HsiB1-HsiE1 crystal structure that in the context of the intact sheath, the N terminus of HsiB1 would need to be accessible for interaction with HsiE1. In contrast, the interaction between HsiB1/TssB and the other sheath component HsiC1/TssC is mostly mediated by a conserved hydrophobic motif in a helix near the C terminus of TssB (24, 48).

Our phylogenetic analysis does not show a strict division into just two classes of T6SSs. Instead, there are mixed clades and isolated branches. For example, we identified four T6SS clusters containing HsiE1 and a ClpV homolog without charged residues in the groove (Fig. 2, *black* and *boxed*), among them *V. parahemeolyticus*. They are notable for two reasons. First, the N termini of their TssB homologs do not display the strict amino acid conservation (Fig. 6*B*, *middle*) seen in T6SSs with a *P. aeruginosa*-type ATPase and a HsiE1 homolog (Fig. 6*B*, *top*). For example, neither Lys^9^ nor Phe^10^, two key residues for the interaction with HsiE1 that we identified in the crystal structure of the HsiB1-HsiE1 complex, are present in those TssB homologs. Second, the cognate HsiE1 homologs lack key residues involved in the TssB-HsiE1 interaction (Fig. 6*B*, *middle*). From the sequence, we predict the absence of a hydrophobic pocket to accommodate a TssB peptide. The structure of VPA1032, the HsiE1 homolog from *V. parahemeolyticus*, bears this out (Fig. 6*A*, *bottom right inset*). This observation suggests that VPA1032 may not interact with its cognate TssB homolog at all, and this HsiE1 homolog may be an evolutionary vestige whose function has been lost.

All four T6SS components studied here are involved in the dynamics of sheath assembly and disassembly. The close evolutionary link among these four proteins suggests a functional connection. At the same time, the evolutionary data suggest that interactions with sheath components and sheath disassembly differ in different T6SS classes. It is possible that the selective pressure imposed by the environment, target cells, and/or competitors has led to the evolution of subtle mechanical differences that dramatically altered the efficacy and speed of the T6SS machine.

**FIGURE 7.**
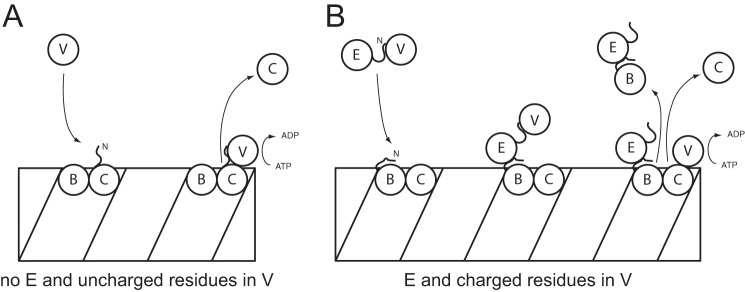
**Speculative model for the role of HsiE/TagJ and ClpV in disassembly of the TssB-TssC sheath.** *A*, in *V. cholerae*, the N terminus of ClpV recognizes the N-terminal helix of the TssC protein VipB and proceeds to disassemble the VipA-VipB sheath. There is no HsiE1 homolog, and the binding groove in the N domain of ClpV contains uncharged residues. *B*, in *P. aeruginosa*, HsiE1 recruits the ATPase ClpV1 to the sheath by recognition of the N terminus of the TssB protein HsiB1. Once at the sheath, ClpV can interact with the TssC protein HsiC1 and starts sheath disassembly. This system is distinguished by the presence of HsiE1 and charged residues in the binding groove of the N domain of ClpV. In both *panels*, *B* denotes the TssB homolog (VipA or HsiB1), *C* denotes the TssC homolog (VipB or HsiC1), and *V* denotes the ClpV homolog (ClpV or ClpV1). In *B*, *E* denotes HsiE1.

In *V. cholerae*, a simple system exists. ClpV directly recognizes the sheath component VipB and drives ATP-dependent disassembly after sheath contraction and toxin ejection (Fig. 7*A*). It is likely that the conformational change associated with contraction (*e.g.* exposing the N terminus of VipB) is the signal for ClpV binding (28). In *P. aeruginosa*, the system is more complex. Free ClpV1 can still interact with HsiC1, but HsiE1 is more likely to recruit ClpV1 to the sheath by recognizing the N terminus of HsiB1 (Fig. 7*B*). It is not clear whether HsiE1 chaperones ClpV1 to the sheath or whether it binds first and provides increased avidity for ClpV1 binding. In either case, proximity of ClpV1 to the sheath would allow the ATPase to recognize and bind HsiC1 and engage in its ATP-dependent disassembly activity.

Because ClpV hexamers bind all along the sheath but in substoichiometric numbers in *V. cholerae* (21), it is interesting to speculate that disassembly of the sheath leads to the accumulation of oligomeric fragments that would either need to be broken down further for reassembly or cleared from the cell. In *P. aeruginosa*, HsiE1-mediated disassembly depends on accessible N termini of HsiB1. It has been suggested in previous studies that the N termini of *V. cholerae* VipA (HsiB) are only accessible at tubule ends (22). The overall similarity of the sheath in *P. aeruginosa* tempts us to speculate on a two-step mechanism, where the initial explosive disassembly mediated by direct interaction between ClpV1 and HsiC1 leads to the production of sheath fragments. These fragments may offer an increased accessibility of HsiB1 N termini and could then be broken down into their components by sequential extraction of HsiC1 upon targeting of ClpV1 to HsiB1 mediated by HsiE1. This model provides ideas to further elucidate the mechanism of sheath disassembly and how it is regulated within the two different classes of T6SSs identified in this study. The T6SS is used by bacteria to fight against each other in a strategic and efficient manner. We can learn from this molecular process and attempt to reroute it to our advantage by designing T6SS-based antimicrobial strategies against bacterial pathogens.

## Footnotes

5 The abbreviations used are:

T6SS: type VI secretion system
r.m.s.: root mean square
BTH: bacterial two-hybrid.
